# Comparative Electrocorticographic Signatures of *S*‑Ketamine and Racemic Ketamine in the Rat Visual Cortex Across Temporal Phases

**DOI:** 10.1021/acsomega.6c02093

**Published:** 2026-07-24

**Authors:** Axell Lins, Luciana Eiró-Quirino, Luana Vasconcelos de Souza, Daniella Rocha Bittencourt, Letícia Cerqueira Duarte, Luiz Fernando Duarte de Andrade Junior, Emile de Almeida Cardoso, Rômulo Augusto Feio Farias, Naoto da Cunha Hayasaki, Fabiano Welter da Silva, Heitor Quaresma Silva, Daniel Bernardo Lopes, Abraão Estevam Lopes, Moisés Hamoy

**Affiliations:** Laboratory of Pharmacology and Toxicology of Natural Products, 37871 Federal University of Pará (UFPA), Belém 66075-110, Brazil

## Abstract

Ketamine enantiomers differ neurophysiologically, yet their specific influence on sensory cortical oscillations remains unclear. Characterizing the electrophysiological signatures of *S*-ketamine (KS) versus racemic ketamine (KSR) in the visual cortex—a region highly sensitive to NMDA blockade—can improve understanding of enantiomer-specific neural modulation and anesthetic depth. This study compared the temporal dynamics of cortical oscillations induced by KS and KSR in rat visual cortices during dissociative anesthesia. Adult Wistar rats received KS (50 mg/kg), KSR (50 mg/kg), or saline. Visual cortex electrocorticographic (ECoG) recordings were obtained for 15 min, segmented into three phases. Power spectral density was calculated across delta to gamma bands (1–40 Hz), and linear power means were compared. KSR produced a deep, stable suppression of oscillatory activity across all frequency bands throughout the recording. Conversely, KS exhibited time-dependent modulation, with a pronounced late-phase increase in delta and theta power, indicating anesthetic superficialization. Furthermore, KS preserved relatively higher overall beta and gamma power, whereas KSR displayed a persistent high-beta elevation (24–28 Hz) typical of NMDA blockade. Ultimately, KSR maintains a stable suppressive pattern, while KS produces late-phase enhancement of slow rhythms. These distinct electrophysiological profiles highlight enantiomer-specific neural dynamics relevant for anesthetic choice and mechanistic modeling.

## 1 Introduction

Ketamine (C_13_H_16_ClNO) is a phencyclidine-derived dissociative anesthetic composed of two enantiomers, *S*-ketamine (KS) and *R*-ketamine (KR). These molecules differ markedly in their pharmacodynamic properties, with KS exhibiting greater affinity as a noncompetitive antagonist of *N*-methyl-d-aspartate (NMDA) receptors—particularly those presynaptically located on glutamatergic neurons—resulting in higher anesthetic potency compared with KR. Clinically, KS is widely employed in human surgical and critical care settings, whereas the racemic formulation (KSR) remains the most commonly used preparation in medical and veterinary procedures requiring anesthesia, analgesia, or rapid dissociation.^1–3^

Beyond its anesthetic properties, ketamine modulates multiple neural systems, notably by increasing dopaminergic transmission in the mesolimbic pathway through NMDA receptor blockade. This inhibition can disinhibit glutamatergic circuits and enhance activation of non-NMDA receptors such as AMPA and kainate, consequently amplifying dopamine release in regions such as the nucleus accumbens.^4–6^ At higher doses, however, widespread glutamatergic inhibition may paradoxically reduce dopaminergic output, producing a state of functional hypoactivity.

Ketamine’s neuropharmacological complexity extends to its effects on cortical dynamics. Studies have shown that ketamine disrupts NMDA- and AMPA-mediated connectivity in frontoparietal networks,^7^ alters high-frequency EEG patterns in humans,^8^ and contributes to neurobiological mechanisms relevant to rapid-acting antidepressant responses.^9^ These actions may vary substantially between enantiomers, as suggested by evidence of differential neuroprotective outcomes^10^ and distinct behavioral or neurochemical responses associated with each compound.^11,12^

Importantly, the visual cortex represents a sensitive region for evaluating NMDA receptor antagonism, given its dense glutamatergic circuitry and its well-characterized oscillatory behavior across delta, theta, alpha, beta, and gamma frequency bands.^13^ Previous work has shown that electrocorticography (ECoG) provides highly informative measures of cortical processing in sensory areas, including primate V1 and rodent models of oscillatory dysfunction.^14,15^ Moreover, ketamine and related NMDA antagonists are known to modulate fast-frequency oscillations, including gamma–band activity, in a dose- and region-dependent manner.^16,17^

Together, these findings underscore the relevance of examining KS and KSR within sensory cortical circuits. However, despite extensive research on ketamine’s molecular and behavioral effects, comparative electrophysiological analyses of KS versus KSR in the visual cortex—and particularly their temporal dynamics during dissociative anesthesia—remain limited. Understanding how each formulation modulates oscillatory patterns may provide critical insights into anesthetic depth, enantiomer-specific neurophysiology, and mechanisms underlying sensory dissociation.

## 2 Materials and Methods

### 2.1 Experimental Animals

A total of 27 adult male Wistar rats, weighing between 220 and 230 g, were used in this study. The animals were obtained from the Animal Facility of the Federal University of Pará (UFPA) and kept in the Laboratory of Pharmacology and Toxicology of Natural Products under controlled conditions: unrestricted access to water and food, constant ambient temperature (23–25 °C), and a 12-h light–dark cycle. The animals underwent a 10-day acclimation period before the experimental procedures. They were housed in pairs in cages measuring 50 × 40 × 30 cm.

All experimental procedures were reviewed and approved by the Ethics Committee on Animal Use (CEUA) under protocol number 3812270225 (ID 002961), in accordance with national legislation governing the care and use of laboratory animals and the ethical guidelines of the National Council for the Control of Animal Experimentation (CONCEA). All experiments were conducted between 07:30 and 11:30 a.m.

### 2.2 Drug Acquisition, Preparation, and Storage

The following chemical substances were used: *R*-ketamine hydrochloride (50%) and *S*-ketamine hydrochloride (50%) obtained from Köing Laboratory (Santana de Parnaíba, SP, Brazil); *S*-ketamine hydrochloride (esketamine) obtained from Cristália Laboratory (Brazil); and 2% lidocaine (local anesthetic) obtained from Hipolabor Laboratory (Sabará, MG, Brazil), used during electrode implantation.

### 2.3 Experimental Design

A ketamine dose of 50 mg/kg was chosen after evaluating the mean time to loss of the righting reflex for ketamine S (51.33 ± 6.4 s) (CI = 46.38–56.29 s) and for ketamine SR (51.56 ± 7.9 s) (CI = 45.42–57.69 s), and recovery of the righting reflex (736.2 ± 120.2 s) (CI = 643.8–828.6) for ketamine S and 832.0 ± 107.2 s (CI = 749.6–914.4 s) for ketamine RS. The visual cortex was recorded for 15 min (900 s).

Animals were randomly assigned to three experimental groups.

(a) **Control group** (*n* = 9): received an intraperitoneal (i.p.) injection of 0.5 mL saline;
(b) **Ketamine*S*/*R*group (KSR)** (*n* = 9): received 50 mg/kg i.p. of racemic ketamine;
(c) **Ketamine-*S*group (KS)** (*n* = 9): received 50 mg/kg i.p. of *S*-ketamine.

ECoG recordings were obtained from the occipital cortex, specifically the secondary visual cortex. Each recording session lasted 15 min following intraperitoneal injection. The recording period was divided into three temporal phases: Phase 1 (0–5 min), Phase 2 (5–10 min), and Phase 3 (10–15 min) (Figure 1).

**Figure 1:**
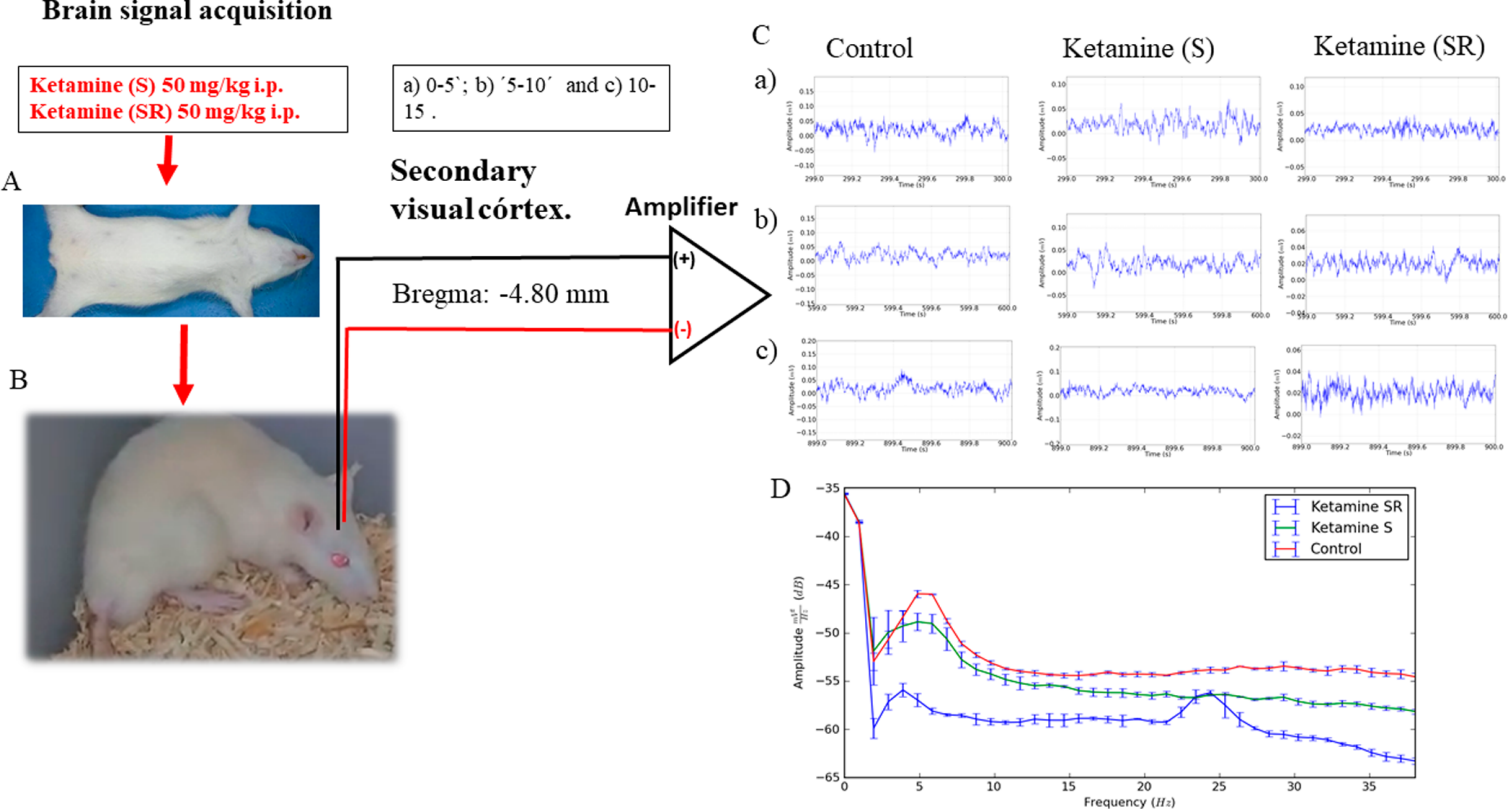
Schematic representation of the procedures used during electrocorticographic (ECoG) data acquisition. Administration of ketamine at 50 mg/kg i.p. (A), followed by electrode connection to a high-impedance amplifier (2000×) for 15 min recording sessions (B). Recordings were analyzed according to the three 5 min phases for the control, *S*-ketamine, and S/R-ketamine groups (C), and variations up to 40 Hz were evaluated through power spectral density analysis (D).

Each phase was analyzed independently to assess variations in oscillatory power across the following frequency bands up to 40 Hz: Delta (1–4 Hz), Theta (4–8 Hz), Alpha (8–12 Hz), Beta (12–28 Hz), and Gamma (28–40 Hz). All data acquisition procedures were performed inside a Faraday cage.

### 2.4 Electrocorticogram (ECoG) Acquisition

Electrodes were surgically implanted at the coordinates bregma −4.80 mm and ±2 mm lateral to target the occipital cortex.^18^ Electrodes were made of stainless steel wire with a diameter of 0.06 mm, covering a recording area of 0.002827 mm^2^ in the visual cortex.

Animals were anesthetized with propofol (100 mg/kg i.p.) until loss of interdigital reflex, followed by infiltration of 0.3 mL of 2% lidocaine at the surgical incision site. Each rat was placed in a stereotaxic frame, and a 1.2 cm incision was made with periosteal retraction to expose the coordinates. Electrodes were positioned on the dura mater and fixed using autopolymerizing dental acrylic.

Following electrode implantation surgery, the animals received a single subcutaneous dose of 1 mg/kg of ketoprofen to control pain and inflammation.

Five days after surgery, electrodes were connected to a high-impedance amplifier (Grass Technologies, P511) monitored by an oscilloscope (Protek 6510). Signals were digitized continuously at 1 kHz using a data acquisition board (National Instruments, Austin, TX, USA), stored on a hard drive, and processed using LabVIEW Express software. The active electrode was placed on the right hemisphere, with the reference electrode on the left (Figure 2A,B).

**Figure 2:**
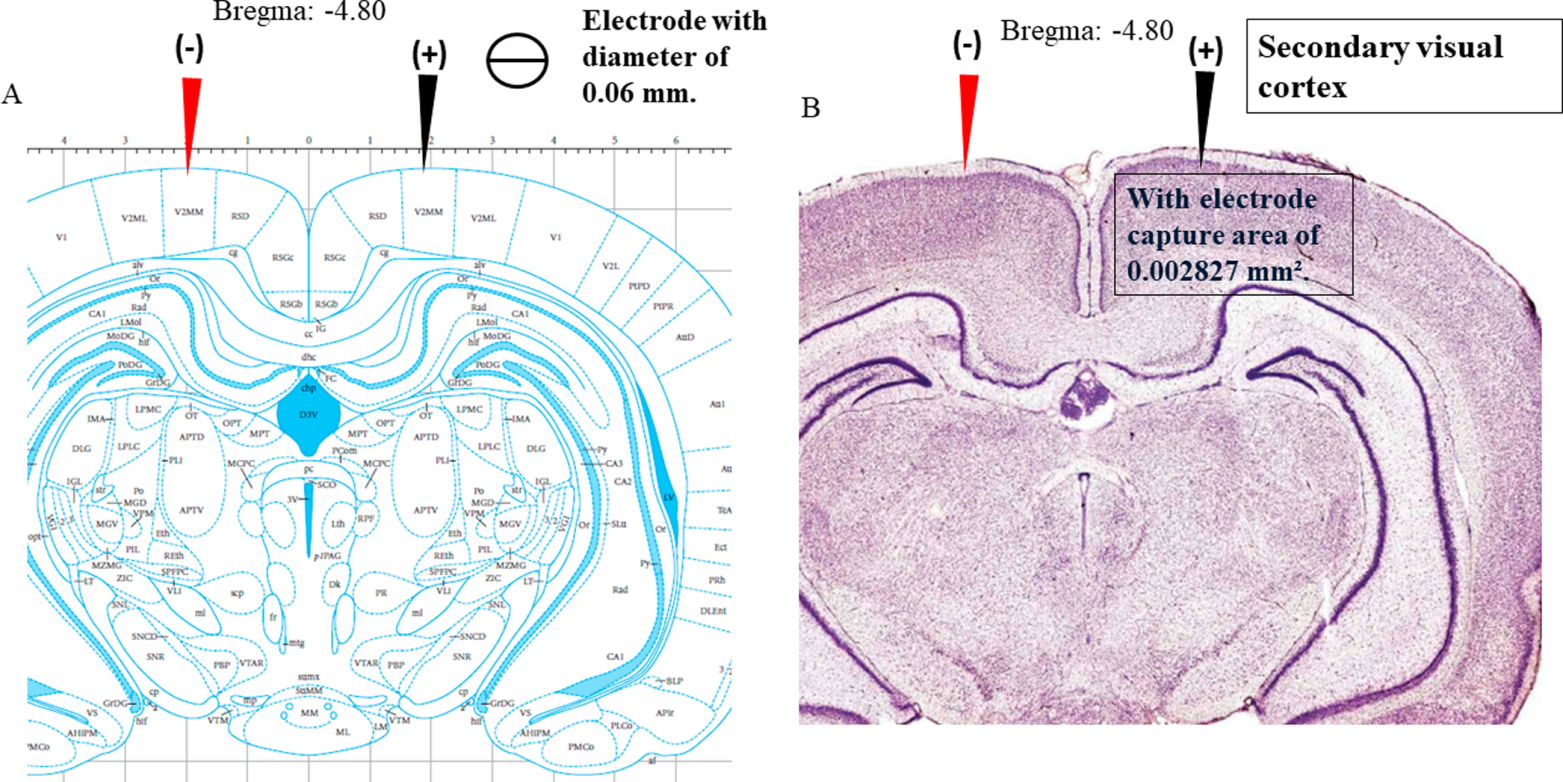
Electrode implantation site positioned at the stereotaxic coordinate bregma −4.80 mm and placed over the dura mater (A). Section of the secondary visual cortex showing electrode placement with a diameter of 0.06 mm and a recording area of 0.002827 mm^2^ (B).

### 2.5 Electrophysiological Data Analysis

To analyze the acquired signals, a custom program was developed in Python 2.7. The Numpy and Scipy libraries were used for mathematical processing, Matplotlib for data visualization, and PyQt4 for the graphical interface. Recordings were analyzed up to 40 Hz, covering all studied oscillatory bands.

### 2.6 Statistical Analysis

After assessing compliance with the assumptions of normality and homogeneity of variances, using the Kolmogorov–Sminov and Levene tests, respectively, both performed in the Python program using the Scipy stats library. Means were compared using a two-way analysis of variance (ANOVA), followed by Tukey’s post-hoc test. Statistical significance was defined as *p < 0.05, **p < 0.01, and ***p < 0.001. Statistical analyses were performed using GraphPad Prism version 8. Data are shown as mean, standard deviation and confidence interval.

## 3 Results

Following administration of KS and KSR at 50 mg/kg i.p., the 15 min recordings showed lower overall power compared with the control group. However, the groups differed from each other in the power spectral density (PSD) profiles: animals treated with KS exhibited higher mean spectral power relative to those treated with KSR. The KS group showed predominance of delta-band oscillations (1–4 Hz), whereas the KSR group demonstrated increased beta–band activity, although still lower than control values (Figure 3A).

**Figure 3:**
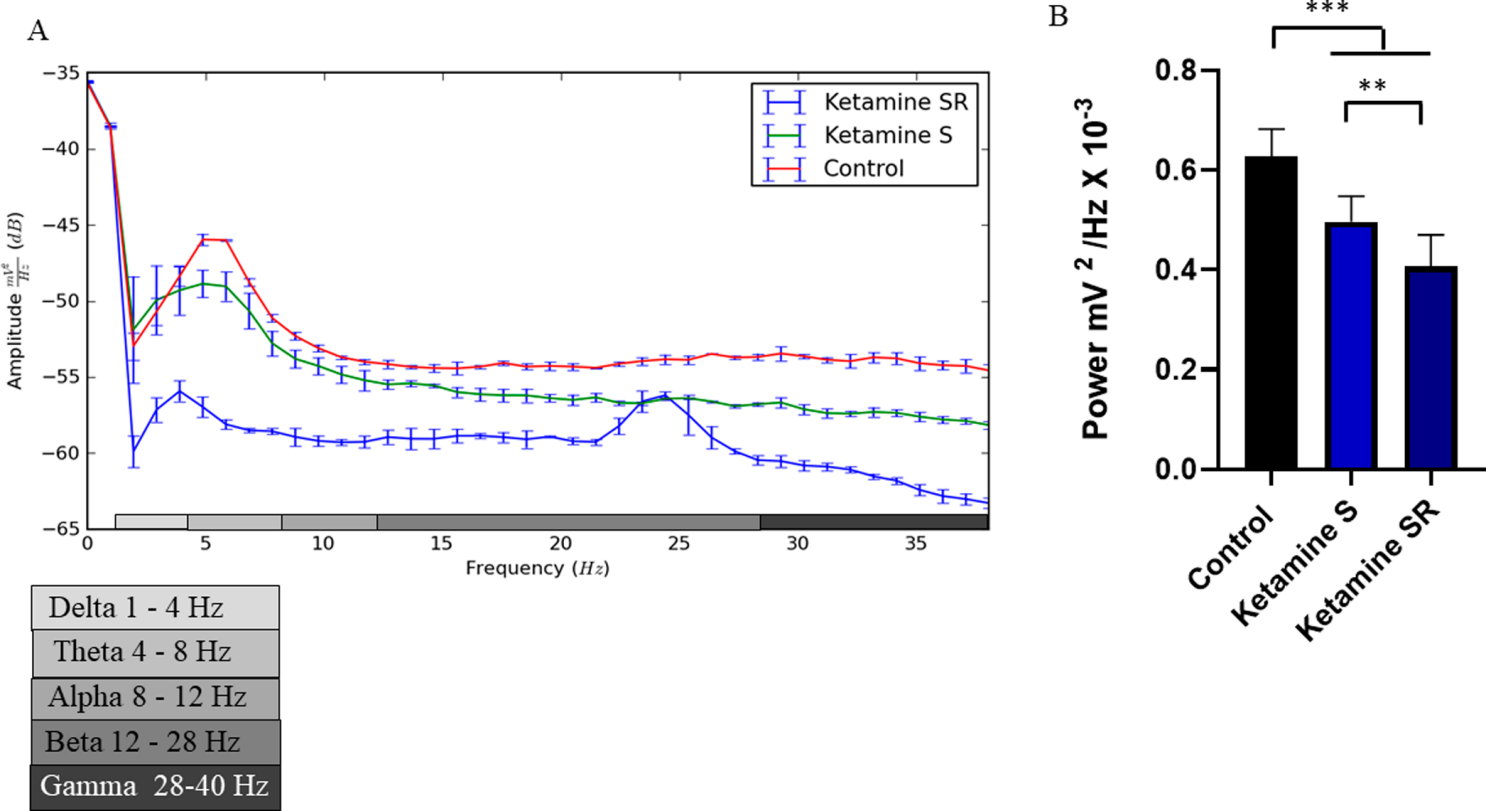
(A) Power spectral density plot showing oscillatory power up to 40 Hz, with identification of delta, theta, alpha, beta, and gamma bands. (B) Linear power values for the control, *S*-ketamine, and S/R-ketamine groups (Two-way ANOVA followed by Tukey’s test; *p* < 0.05, *p* < 0.01, *p* < 0.001; *n* = 9).

When analyzing mean linear power, the control group (0.626 ± 0.058 mV^2^/Hz × 10^–3^) (CI = 0.582–0.669) showed higher values than both ketamine-treated groups. The KS group (0.496 ± 0.0513 mV^2^/Hz × 10^–3^) (CI = 0.457–0.535) displayed higher mean power compared with the KSR group (Figure 3B).

During the temporal-phase evaluation of KS and KSR, the control group exhibited a stable electrocorticographic trace throughout the 15 min recording, with an amplitude of 0.05 mV, visible in the 1-s magnifications at the end of each phase. In the PSD plot up to 40 Hz, delta, theta, and alpha oscillations predominated consistently across all phases (Figure 4A).

**Figure 4:**
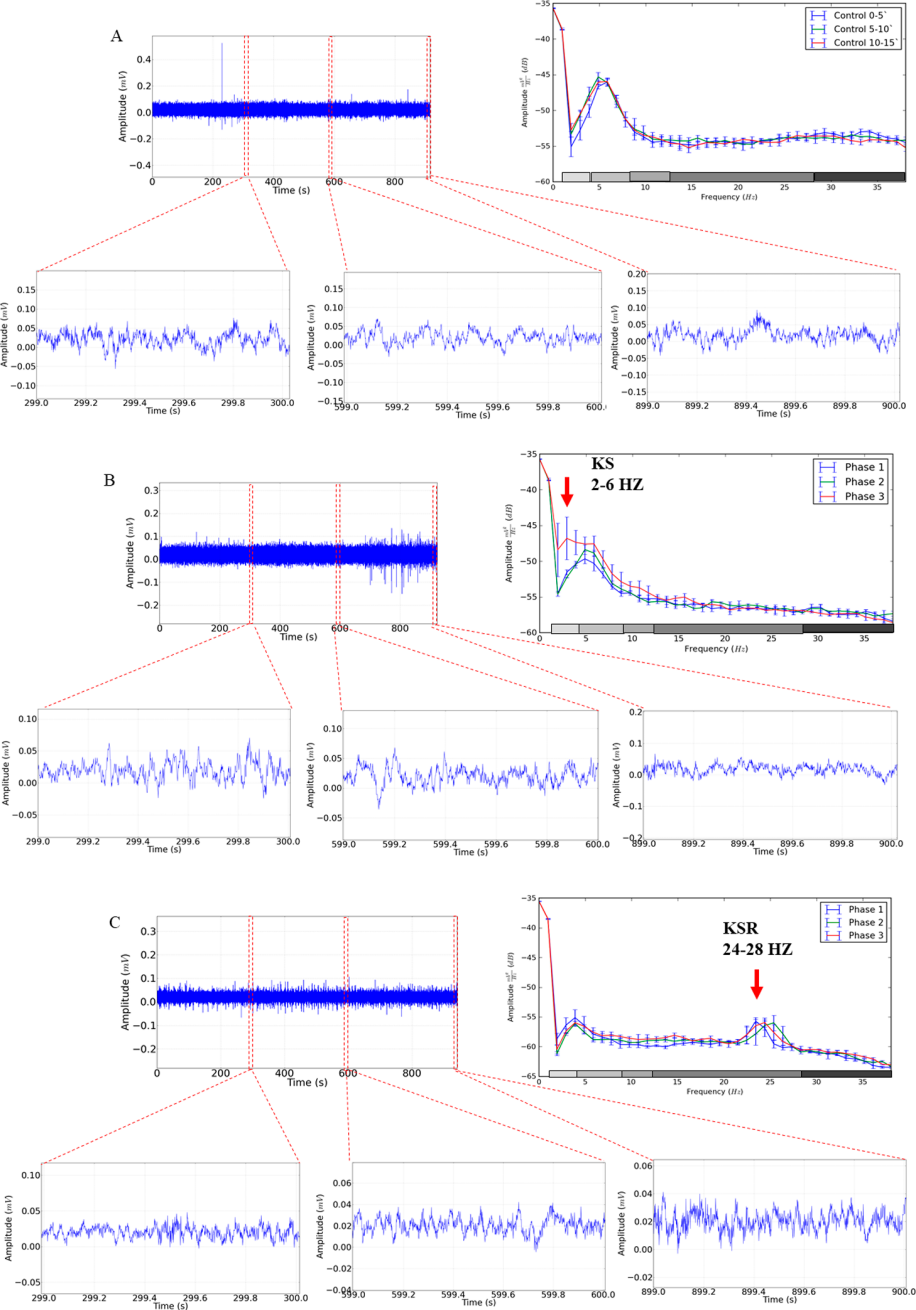
Fifteen-minute electrocorticographic trace from the visual cortex with 1-s magnifications at the end of each 5 min interval (highlighted with red dashed boxes). Power spectral density (PSD) plots up to 40 Hz for the following groups: Control (A), *S*-ketamine (B), and *S*/*R*-ketamine (C).

After KS administration, the ECoG trace became more irregular, particularly during phase 3 (corresponding to 600–900 s). The amplitude remained at 0.05 mV during the first 10 min, increasing to 0.1 mV in the third phase. PSD analysis showed a similar power distribution in phases 1 and 2, but in phase 3 there was a power increase in the 2–6 Hz range, leading to higher delta and theta oscillatory means (Figure 4B).

In the KSR-treated group (50 mg/kg i.p.), recordings showed an irregular trace with spaced low-amplitude spikes. The phases displayed predominant amplitudes around 0.04 mV, with variations up to 0.1 mV as shown in the magnified segments. The PSD distribution revealed similar power profiles across all frequencies up to 40 Hz, but with a notable increase in the 24–28 Hz range, making beta oscillations predominant relative to other frequencies (Figure 4C).

When comparing phases 1, 2, and 3, the control group showed a mean linear power of 0.626 ± 0.056 mV^2^/Hz × 10^–3^ (CI = 0.582–0.669), similar only to KS phase 3 (*p* = 0.998), but greater than all other treated groups. KS phase 1 (0.476 ± 0.072 mV^2^/Hz × 10^–3^) (CI = 0.420–0.532) was similar to KSR phase 1 (*p* = 0.115), and KS phase 2 (0.462 ± 0.084 mV^2^/Hz × 10^–3^) (CI = 0.397–0.527) was similar to KSR phase 2 (*p* = 0.155). KS phase 3 (0.608 ± 0.071 mV^2^/Hz × 10^–3^) (CI = 0.553–0.664) was significantly higher than KSR phase 3 (Figure 5A).

**Figure 5:**
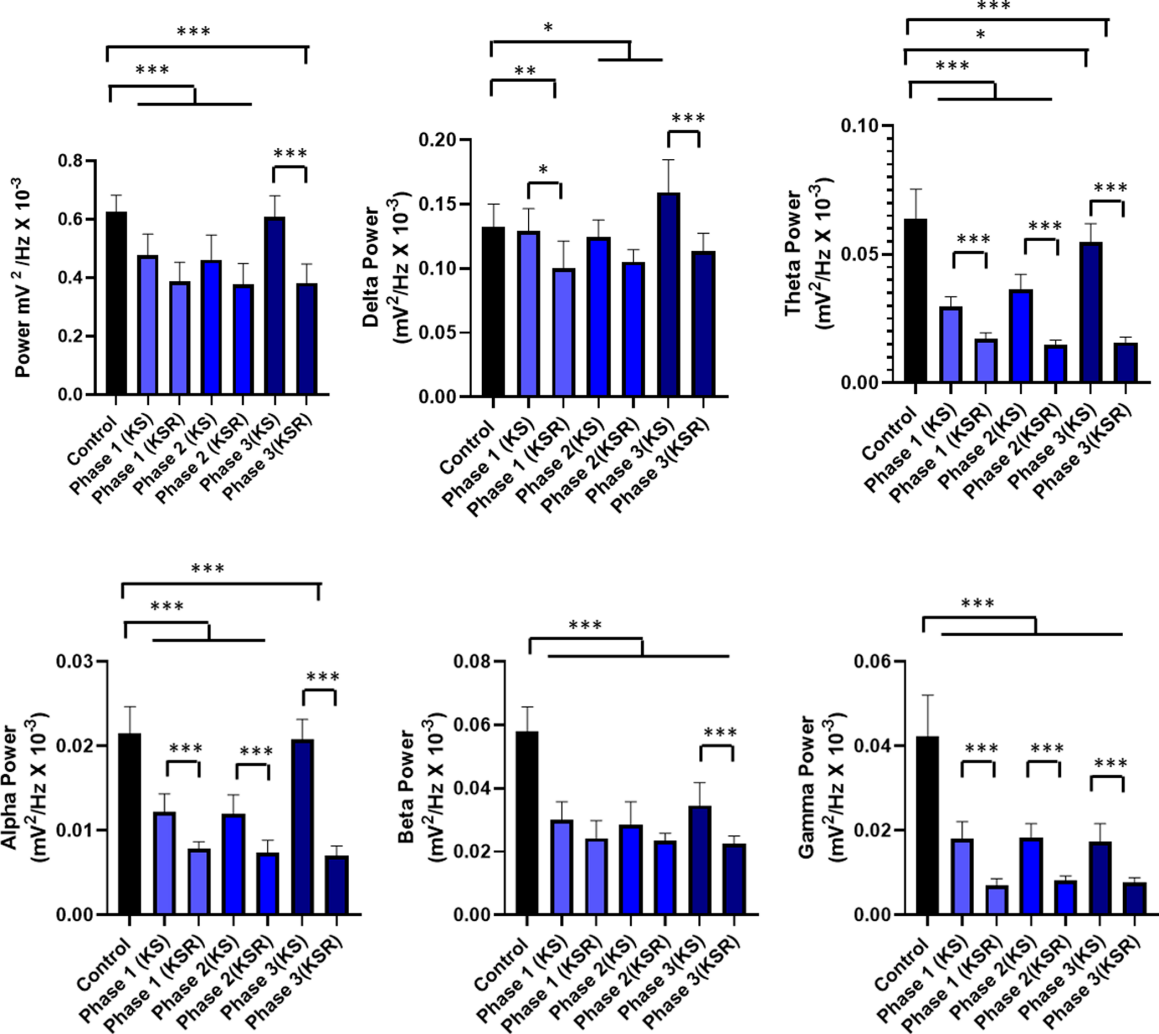
Mean linear power across 5 min temporal phases (0–300 s, 300–600 s, and 600–900 s) following ketamine administration. Distribution of power values across delta, theta, alpha, beta, and gamma frequency bands (Two-way ANOVA followed by Tukey’s test; *p* < 0.05, *p* < 0.01, *p* < 0.001; *n* = 9).

For delta oscillations, the control mean (0.132 ± 0.0172 mV^2^/Hz × 10^–3^) (CI = 0.118–0.146) was similar to KS phase 1 (*p* = 0.999), KS phase 2 (*p* = 0.969), and KS phase 3 (*p* = 0.285). KS phase 1 (0.129 ± 0.0175 mV^2^/Hz × 10^–3^) (CI = 0.115–0.142) showed higher values than KSR phase 1. KS phase 2 (0.124 ± 0.0134 mV^2^/Hz × 10^–3^) (CI = 0.139–0.178) was similar to KSR phase 2 (*p* = 0.238). KS phase 3 (0.159 ± 0.025 mV^2^/Hz × 10^–3^) was higher than KSR phase 3 (Figure 5B).

For theta oscillations, the control group (0.066 ± 0.0116 mV^2^/Hz × 10^–3^) (CI = 0.0548–0.0727) showed higher values than all KS and KSR phases. KS phases 1 (0.0296 ± 0.0038 mV^2^/Hz × 10^–3^) (CI = 0.026–0.032), 2 (0.0363 ± 0.0058 mV^2^/Hz × 10^–3^) (CI = 0.0318–0.0409), and 3 (0.0547 ± 0.0072 mV^2^/Hz × 10^–3^) (CI = 0.0491–0.0603) all showed higher values than their respective KSR phases (Figure 5C).

For alpha oscillations, the control group (0.0215 ± 0.0031 mV^2^/Hz × 10^–3^) (CI = 0.0190–0.0239) was similar only to KS phase 3 (*p* = 0.988), but superior to all other groups. KS phase 1 (0.0125 ± 0.0021 mV^2^/Hz × 10^–3^) (CI = 0.0106–0.0138) was higher than KSR phase 1; KS phase 2 (0.0120 ± 0.0022 mV^2^/Hz × 10^–3^) (CI = 0.0102–0.0137) was higher than KSR phase 2; and KS phase 3 (0.0207 ± 0.0023 mV^2^/Hz × 10^–3^) (CI = 0.189–0.0226) was higher than KSR phase 3 (Figure 5D).

For beta oscillations, the control group (0.0579 ± 0.0077 mV^2^/Hz × 10^–3^) (CI = 0.0519–0.0639) showed higher values than all ketamine-treated groups. KS phase 1 (0.0300 ± 0.0057 mV^2^/Hz × 10^–3^) (CI = 0.0255–0.0344)­was similar to KSR phase 1 (*p* = 0.377). KS phase 2 (0.0285 ± 0.0116 mV^2^/Hz × 10^–3^) (CI = 0.0229–0.0340)­was similar to KSR phase 2 (*p* = 0.545). KS phase 3 (0.0340 ± 0.0072 mV^2^/Hz × 10^–3^) (CI = 0.0290–0.0401) was higher than KSR phase 3 (Figure 5E).

For gamma oscillations, the control group (0.0423 ± 0.0097 mV^2^/Hz × 10^–3^) (CI = 0.0348–0.0498) exhibited higher power than all ketamine-treated groups. KS phases 1 (0.0180 ± 0.0040 mV^2^/Hz × 10^–3^) (CI = 0.0149–0.0212), 2 (0.0183 ± 0.0033 mV^2^/Hz × 10^–3^) (CI = 0.0157–0.02084), and 3 (0.0172 ± 0.0011 mV^2^/Hz × 10^–3^) (CI = 0.0139–0.0206) showed consistently higher values than KSR in the corresponding phases (Figure 5F).

## 4 Discussion

The present study provides a relevant contribution by comparing, with high temporal resolution, the electrophysiological effects of *S*-ketamine (KS) and racemic ketamine (KSR) in the visual cortex of Wistar rats. Although both formulations are widely used in anesthetic practice and extensively investigated for their neuropsychiatric actions, important gaps remain regarding how each enantiomer modulates cortical activity over time. By segmenting ECoG recordings into three temporal phases and analyzing delta, theta, alpha, beta, and gamma frequency bands, this study demonstrates distinct neurophysiological signatures induced by each formulation, reinforcing meaningful differences in the depth and stability of dissociative anesthesia. Although ketamine S and ketamine SR have distinct pharmacokinetic profiles, with ketamine S being more rapidly metabolized,^30^ behavioral data have shown that at a dose of 50 mg/kg i.p., induction and recovery are very similar in rats.

Our results show that KS produced a more dynamic modulation pattern, characterized by an increase in low-frequency oscillations (delta and theta) during phase 3, indicating a shallowing of the anesthetic plane and potential partial re-establishment of thalamo-cortical synchrony. This finding aligns with the known role of slow oscillations as classic markers of anesthetic transitions, reflecting global neuronal integration and fluctuations in the level of consciousness. Previous studies have shown that ketamine can disrupt functional connectivity and induce frequency-specific changes in cortical networks.^7,19,20^ However, the predominance of delta and theta observed in the KS group during the late phase suggests a transition pattern distinct from that induced by the racemic formulation.

In contrast, KSR produced deeper and more consistent electrophysiological suppression across the three phases, with reduced power in nearly all frequency bands and a modest relative increase in beta (24–28 Hz). This profile is consistent with studies demonstrating that racemic ketamine tends to produce more sustained cortical hypoactivity, likely due to the slower pharmacokinetics of the R-enantiomer, which prolongs network depression.^6^ The stability of spectral power observed under KSR reinforces its capacity to maintain deeper anesthetic states, as reported by Midega et al.,^21^ clearly differing from the more fluctuating profile observed with KS.

The differential modulation of fast-frequency bands also deserves attention. Although both formulations significantly reduced gamma power relative to controls, KS preserved slightly higher beta and gamma activity compared with KSR, even though both remained globally suppressed. The literature indicates that fast oscillations reflect cortical disinhibition, AMPA-mediated glutamatergic modulation, and transient reorganization of neural networks.^14,16,17,22^ Thus, the lower fast-frequency activity observed under KSR reinforces its more pronounced suppressive profile, consistent with deeper anesthesia. The small beta elevation observed in KSR does not contradict this interpretation; the increase was small and restricted to a narrow range, as described in studies of paradoxical oscillatory states under ketamine.^8^

A major portion of our findings aligns with the “paradoxical” pattern described by Akeju et al.,^23^ in which ketamine induces alternation between high-frequency bursts (beta/gamma) and brief periods of slow oscillations, generating a state of functional incoherence rather than the diffuse cortical suppression typical of GABAergic agents. In the present study, this signature was more evident in the KSR group, which showed profound reductions in slow bands (delta and theta) along with a modest relative increase in beta, suggesting thalamo–cortical desynchronization similar to patterns observed in humans.

Furthermore, the computational model proposed by Elie Adam et al.^24^ reinforces that NMDA receptor blockade disrupts the generation of normal slow rhythms, leading to predominance of irregular high-frequency firing and functional disorganization. This interpretation is consistent with the stronger suppression of slow oscillations observed under KSR, indicating a deeper dissociative state than that produced by KS.

The contribution of the R enantiomer to this profile becomes particularly evident when considering that KS has a higher affinity for the NMDA receptor and produces rapidly onset dissociative effects,^12^ while the *R* enantiomer exhibits slower elimination and may prolong states of cortical disorganization. This helps explain why, despite its lower pharmacological potency, KSR produced greater suppression of cortical oscillations, as also suggested by Pfenninger et al.^25^ and corroborated by clinical and experimental findings.

Although gamma oscillations were not preserved in either group—a phenomenon expected at the 50 mg/kg dose—it remains important to contextualize their physiological role. Gamma bands are associated with motor, linguistic, perceptual, and visual functions,^26–28^ and their reduction is frequently observed under states of low cortical excitability or NMDA antagonismo.^29^ Studies such as Hiyoshi et al.^16^ also show that higher ketamine doses suppress gamma activity, which explains the low expression of this band in both groups here, demonstrating that both ketamine S and ketamine SR decrease the power of gamma oscillations.

The literature also suggests that reduced gamma activity can reflect structural or functional alterations in pyramidal neurons,^13^ and that racemic ketamine may exert neuroprotective effects in specific conditions such as spinal cord injury, although these effects depend on injury stage.^10^ While these mechanisms were not evaluated directly in this study, they contextualize the suppression of fast-frequency bands observed in our recordings.

Finally, studies involving psychosis models, such as Welle,^15^ indicate that NMDA antagonists can induce gamma desynchronization in V1, similar to what we observed, reinforcing that ketamine—particularly in racemic formulations and at higher doses—tends to disrupt fast rhythms in visual cortical circuits. Although our logs reflect the V2 signature, a similar pattern was observed.

Taken together, our findings indicate that KS and KSR produce distinct electrophysiological patterns in the visual cortex of rats. KSR induced deeper, more stable, and more global oscillatory suppression, whereas KS generated greater variability, including late-phase increases in delta and theta compatible with anesthetic lightning. These results support the interpretation that the racemic formulation is more suitable for maintaining deep anesthetic states, as emphasized in the literature, whereas KS exhibits more dynamic and transient signatures aligned with its faster and more potent pharmacokinetic profile. However, our work has limitations because only one dose of stamina was evaluated without comparison to other equipotent anesthetics, the recordings were made only in one region of the brain (secondary visual cortex), no intraindividual baseline analysis was performed, and the recording time window was too short for complete analysis. Furthermore, no activity of ketamines at NMDA receptors was performed to quantify their activity.

## 5 Conclusions

In conclusion, *S*-ketamine and racemic ketamine produce distinct electrophysiological signatures in the secondary visual cortex of rats, reflecting different patterns of cortical oscillatory power modulation. The racemic formulation generated a more pronounced suppression throughout the three temporal phases. In contrast, *S*-ketamine produced a marked increase in delta and theta power during the third phase, suggesting greater superficialization of anesthesia. These findings suggest that racemic ketamine induces more stable anesthetic planes in rats, facilitating its use for chemical restraint and/or surgical procedures.

## Data Availability

All data generated or analyzed during this study are available in the public repository at the following link: https://drive.google.com/file/d/1MrJjR7FDyJ6TfcWDFkNVgg8mHmmJqsH9/view?usp=sharing. The data sets support the findings of this study and include the original electrophysiological and behavioral data.
